# Effect of the learning curve on survival after laparoscopic liver resection for colorectal metastases

**DOI:** 10.1093/bjsopen/zrac020

**Published:** 2022-04-05

**Authors:** Hiba Shanti, Rakesh Raman, Saurav Chakravartty, Ajay P. Belgaumkar, Ameet G. Patel

**Affiliations:** Institute of Liver Studies, King’s College Hospital, London, UK; Kent Oncology Centre, Kent and Canterbury Hospital, Canterbury, UK; Department of Surgery, St Richard’s Hospital, Chichester, UK; Department of Surgery, Surrey and Sussex Healthcare NHS Trust, Redhill, UK; Institute of Liver Studies, King’s College Hospital, London, UK

## Abstract

**Background:**

Laparoscopic liver resection (LLR) is a highly demanding procedure with great variability. Previously published randomized trials have proven oncological safety of laparoscopic liver resection (LLR) as compared to open surgery. However, these were started after the learning curve (LC) was established. This leaves the question of whether the LC of LLR in the early laparoscopic era has affected the survival of patients with colorectal liver metastasis (CRLM).

**Methods:**

All consecutive LLRs performed by a single surgeon between 2000 and 2019 were retrospectively analysed. A risk-adjusted cumulative sum (RA-CUSUM) chart for conversion rate and the log regression analysis of the blood loss identified two phases in the LC. This was then applied to patients with CRLM, and the two subgroups were compared for recurrence-free (RFS) and overall survival (OS). The analysis was repeated with propensity score-matched (PSM) groups

**Results:**

A total of 286 patients were included in the LC analysis, which identified two distinct phases, the early (EP; 68 patients) and the late (LP; 218 patients) phases. The LC was applied to 192 patients with colorectal liver metastasis (EPc, 45 patients; LPc, 147 patients). For patients with CRLM, R0 resection was achieved in 93 per cent: 100 per cent in the EPc group and 90 per cent in the LPc group (*P* = 0.026). Median OS and RFS were 60 and 16 months, respectively. The 5-year OS and RFS were 51 per cent and 32.7 per cent, respectively. OS (hazard ratio (h.r.) 0.78, 95 per cent confidence interval (c.i.) 0.51 to 1.2; *P* = 0.286) and RFS (h.r. 0.94, 95 per cent c.i. 0.64 to 1.37; *P* = 0.760) were not compromised by the learning curve. The results were replicated after PSM.

**Conclusion:**

In our experience, the development of a laparoscopic liver resection programme can be achieved without adverse effects on the long-term survival of patients with CRLM.

## Teaser

This paper aimed to evaluate the effect of the learning curve of laparoscopic liver resection on the long-term survival of patients with colorectal liver metastasis. In this study of 286 patients, RA-CUSUM of the conversion rate and log regression analysis of blood loss estimated a learning curve of 68 cases to attain proficiency in laparoscopic liver resection. When this was applied to patients with colorectal liver metastasis, the overall and recurrence-free survival rates were not compromised by the learning curve.

## Introduction

Colorectal cancer (CRC) is the second leading cause of cancer death in Western Europe and the USA. The liver is the main organ for metastases, with 15 to 25 per cent of patients presenting with synchronous disease. Approximately 70 per cent of patients with CRC will develop liver metastasis^1^. Surgical resection remains the cornerstone of potentially curative therapy and is the key determinant of survival.

The benefits of laparoscopic abdominal surgery have been well demonstrated and include reduced postoperative pain and analgesic requirements, quicker recovery, fewer wound complications, and shorter duration of hospital stay^1–3^. This has resulted in an increased uptake of laparoscopic liver resections (LLR) over the last two decades. However, this had a prolonged development. After Gagner introduced LLR in 1992^4^, It was not until 2004 that the first series with more than 10 laparoscopic major liver resections was published^5^. Furthermore, a multicentre study by Allard *et al*.^6^ in 2015 showed that laparoscopy was only used in 176 (6.7 per cent) patients out of a total of 2620 patients treated for CRLM. This lag time in the establishment of LLR was attributed to the steep learning curve (LC) due to technical complexity and considerable variability in LLR, as well as the initial caution exhibited by surgeons regarding the oncological safety of laparoscopic surgery. Previously published studies investigating the LC in LLR showed that 45 to 75 cases are required before achieving proficiency^7–9^.

The aim of this study was to assess whether the learning curve of LLR in the early laparoscopic era has affected the survival of patients with colorectal liver metastasis (CRLM).

## Methods

Between July 2000 and December 2019, all LLRs that were performed by a single hepatopancreatic-biliary surgeon at the Institute of Liver Studies at King’s College Hospital, London, UK, were retrospectively collected from a prospectively developed database.

From the cohort of LLR, the LC was first established and analysed. This generated two phases of the LC: the early phase (EP) and the late phase (LP). These were compared for patient characteristics, extent of resection, and perioperative outcomes.

The outcome of the LC was then applied to all patients with CRLM. Two subgroups were generated: the early phase (EPc) and the late phase (LPc). EPc and LPc were compared for patient characteristics, tumour staging, and oncological and survival outcomes.

Owing to the expected degree of bias in the historical comparison between EPc and LPc, a propensity score match was performed, and the matched groups EPcpsm and LPcpsm were eventually compared for oncological outcomes.

### Data collection and definitions

Data collected included sex, age, ASA grade, tumour size and location, the extent of surgical resection, operating time, estimated intraoperative blood loss, conversion to open surgery, duration of hospital stay, and postoperative complications. The latter were classified according to the Dindo-Clavien system^10^. Perioperative mortality was defined as death within 90 days of the operation or within the same hospital admission. For patients with CRLM, further oncological data were obtained, including details of the primary tumour, number and size of the liver metastases, recurrence, repeated resections or interventions, chemotherapy, and survival. All patients underwent a surveillance protocol to detect recurrence.

R0 resection was defined as more than 1 mm from the resection margin. The two endpoints for survival evaluation were overall survival (OS) and recurrence-free survival (RFS). Time of survival was calculated from the date of hepatectomy until the date of one of the following events: death for OS, and death or first recurrence (intrahepatic/extrahepatic) for RFS. Patients who did not have the event at the end of the follow-up were censored.

### Establishment of the learning curve

The entire LLR cohort was used to establish the LC based on two parameters, blood loss and conversion, as suggested by previous studies^8,9,11^. This analysis was performed using a different methodology for each parameter.

Estimated intraoperative blood loss was plotted for each case against the time sequence; a curve was generated using log regression analysis. The estimation of the LC was based on the transition phase between the early sharp drop in blood loss to a later, more gradual decline over time.

For the conversion rate, the risk-adjusted cumulative log-likelihood ratio statistic (RA-CUSUM) was used; every conversion was considered as failure. The graph starts at 0 and is incremented by 1–*s_i_* for failure and decremented by *s_i_* for success. The value of *s_i_* is defined by the predicted risk of failure (conversion) for operation *i*. The graph moves upward if the failure rate increases and downward if it decreases. Resections were categorized according to the IWATE score (proposed by the International Consensus Conference on Laparoscopic Liver Resection in Japan) into four grades of difficulty based on tumour size, position, liver function, and extent of resection^12^. The predicted risk for conversion was estimated based on the IWATE score and the literature-predicted risk of conversion, which ranges between 4 per cent and 17 per cent^8,9,11,13^.

The LCs established using these two methodologies, blood loss and conversion, were correlated and used to divide the entire cohort into two groups—EP and LP.

### Propensity score matching and statistical analysis

Propensity analysis was calculated using logistic regression to obtain a propensity score for each patient with the covariates, including tumour characteristics affecting long-term outcomes. The covariates included maximum tumour dimensions (less than 5 cm or 5 cm or more); number of liver lesions (solitary or multiple); distribution in the liver (bi- or unilobar), the timing of liver metastasis (synchronous or metachronous); lymph node status of the primary tumour; and the presence of extrahepatic disease. Patients in EPc were matched 1:1 with patients in LPc group. We applied the nearest neighbour matching within a calliper of width equal to 0.2. All variables were balanced with an average standardized mean difference of covariates in the postmatch sample of 0.049.

The results are expressed as median (range) for continuous variables, or as number (percentage) for categorical variables. The differences in distribution tests were analysed with χ^2^ tests or Fisher’s exact test for categorical variables and Wilcoxon score tests for continuous variables. The threshold for statistical signiﬁcance was set at a *P* value less than 0.05. OS and RFS were analysed using the Kaplan–Meier method and the Cox proportional hazards model. A *P* value less than 0.05 was considered significant. Statistical analysis was performed using Stata Release 16 (StatCorp, College Station, TX, USA).

## Results

### Analysis of the entire cohort

Two hundred and eighty-six patients underwent LLR. The patients had a median age of 63 (range 22 to 87) years and there were more women than men (154 women; 53.8%); the majority had malignant disease (79 per cent). LLR included major resections (27 per cent), anatomical resections (43 per cent), and non-anatomical resections (57 per cent). The IWATE score ranged evenly between I and IV (*Table 1*). Median operative time was 190 (range 60 to 840) minutes. The overall conversion rate was 9 per cent (27 patients). Postoperative complications occurred in 15 per cent (43 patients), with a major complication (defined as Clavien-Dindo class III or higher) in 6.9 per cent (20 patients). The median duration of hospital stay was 5 days, with no 90-day or inpatient mortality.

**Table 1:** Patients’ characteristics, operative, and perioperative outcomes

	EP (*n* = 68)	LP (*n* = 218)	*P*
**Patient characteristics**			
Median (range) age (years)	61.5 (27–77)	64 (22–87)	0.670
Age > 70 years	14 (21)	66 (30.2)	0.120
Male sex	31 (46)	101 (46.3)	0.914
ASA grade			
I	20 (29)	51 (23.3)	0.314
II	42 (62)	136 (62.3)	0.929
III	6 (9)	31 (14.2)	0.201
Malignant lesions	52 (77)	174 (79.8)	0.511
**Surgical procedure**			
Non-anatomical/ metastasectomy	16 (24)	89 (40.8)	0.006
Bi-segmentectomy	15 (22)	31 (14.2)	0.164
Tri-segmentectomy	1 (1)	10 (4.6)	0.185
Left lateral sectionectomy	22 (32)	36 (16.5)	0.013
Right hepatectomy (+extended)	12 (18)	30 (13.7)	0.405
Left hepatectomy (+extended)	6 (9)	21 (9.6)	0.134
Additional wedge resection	6 (9)	13 (5.9)	0.531
Proximity to vascular structures	9 (13)	60 (27.8)	0.020
Major resection	19 (28)	68 (31.1)	0.612
**IWATE level**			.27
I	10 (15)	48 (22.0)	0.190
II	24 (35)	61 (27.9)	0.249
III	19 (28)	65 (29.8)	0.766
IV	15 (22)	44 (20.1)	0.738
**Perioperative outcomes**			
Median (range) duration of surgery (min)	240 (90–840)	150 (60–660)	0.001
Median (range) intraoperative blood loss (ml)	200 (100–5000)	100 (20–5000)	0.001
Pringle’s manoeuvre	0 (0)	41 (18.8)	0.000
Conversion	13 (19)	14 (6.4)	0.002
Postoperative complications	16 (24)	27 (12.4)	0.025
Clavien-Dindo above III	8 (12)	12 (5.5)	0.077
Bleeding	12 (18)	9 (4.1)	0.000
Reoperation	4 (6)	1 (0.5)	0.003
Blood transfusion	15 (22)	19 (8.7)	0.003
Bile leak	1 (2)	7 (3.2)	0.441
Median duration of hospital stay (days)	7 (2–147)	5 (1–112)	0.000

Data are *n* (%) unless otherwise stated. EP, early phase; LP, late phase.

### Establishment of the learning curve

**Fig. 1:**
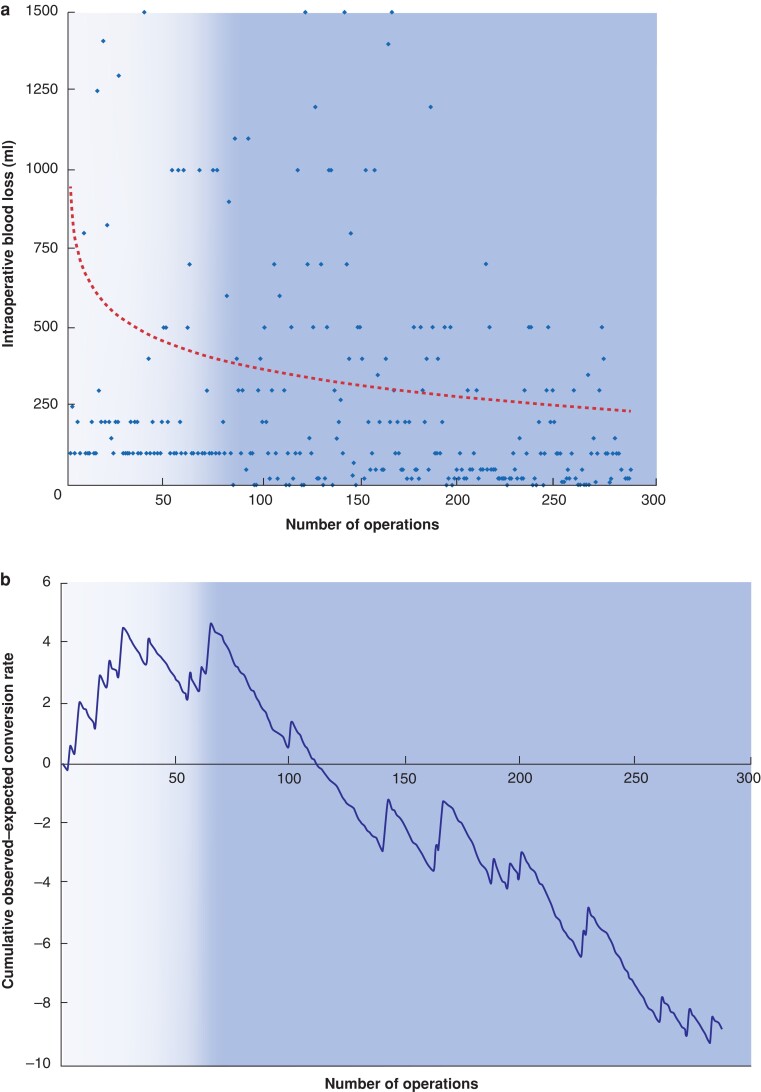
Establishment of the learning curve

The median intraoperative blood loss was 100 (range 20 to 5000) ml, with a transfusion rate of 12 per cent. Intraoperative blood loss showed a downward trend over time, with no clear breakpoint. A sharp inflexion at 50 to 70 cases was found, before turning into a steady curve (*Fig. 1*).

**a** Logarithmic regression learning curve for intraoperative blood loss. **b** Risk-adjusted cumulative log-likelihood ratio statistic (RA-CUSUM) analysis of conversion rate.

**Fig. 2:**
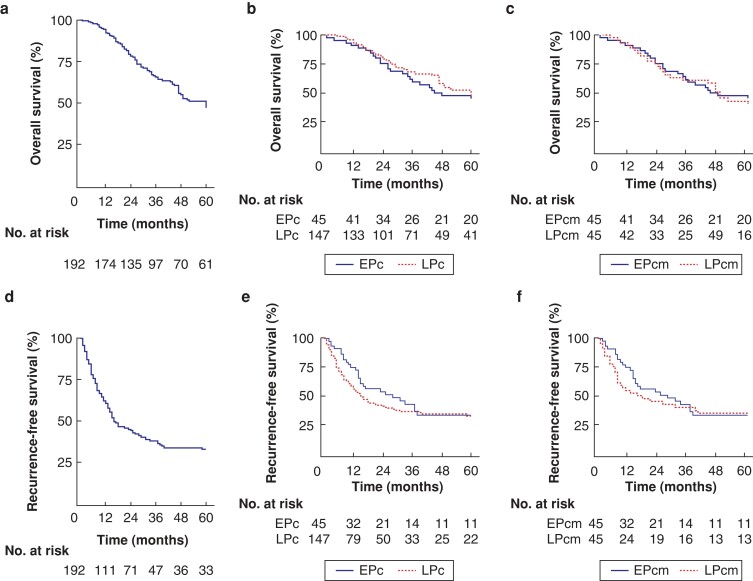
Overall and regression-free survival for patients with colorectal liver metastatis (CRLM)

The RA-CUSUM for the conversion rate showed a peak at 68 cases, followed by a decline in conversions (*Fig. 2*). Combining the results from the RA-CUSUM and the blood loss log curve, the EP (68 patients) and the LP (218 patients) were identified, with the split occurring in mid-2007.

**a** Overall survival (OS) of the whole cohort of CRLM. **b** OS comparison between the early-phase (EPc) and late-phase (LPc) cohorts. **c** OS of the propensity score-matched groups. **d** Recurrence-free survival (RFS) of the whole cohort of patients with CRLM. **e** RFS comparison between EPc and LPc. **f** RFS of the propensity score-matched groups.

### Comparison of patients’ characteristics and perioperative outcomes between the two phases

Patients in the LP were older, with more comorbid conditions. However, this was not statistically significant. The distributions of IWATE difficulty grades were similar between the two phases, although LP had significantly more non-anatomical metastectomies. The LP had significantly less perioperative blood loss, a lower transfusion rate, a lower overall complication rate, a shorter operating time, and a shorter duration of hospital stay (*Table 1*).

The conversion rate dropped significantly in the LP from 13 of 68 (19.1 per cent) to 14 of 218 (6.4 per cent; *P* =   0.002). The reason for a higher conversion in the EP was bleeding (seven of 13; 54 per cent) and oncological inadequacy or resection of other organs (six of 13; 46 per cent). In the LP, one of 14 (7 per cent) conversions was for bleeding, while 13 (93 per cent) were for oncological reasons, mainly related to direct invasion into adjacent organs or for threatened margin clearance. All reoperations were for postoperative bleeding.

### Oncological outcomes analysis

One hundred and ninety-two patients had LLR for CRLM. Of these, 45 cases were performed in the EP (EPc) and 147 in the LP (LPc). Both groups had similar patient characteristics and ASA grade. The LPc had more patients with lymph node-positive primary tumours. Other tumour and primary disease characteristics were similar between the two groups (*Table 2*).

**Table 2:** Characteristics of patients with colorectal liver metastasis and oncological data

	EPc (*n* = 45)	LPc (*n* = 147)	*P*
**Patient characteristics**			
** **Median (range) age (years)	65 (27–77)	66 (23–87)	0.496
Age > 70 years	11 (24)	49 (33.3)	0.299
Male sex	26 (58)	78 (53.1)	0.581
ASA			
I	8 (18)	28 (19.0)	0.848
II	33 (73)	99 (67.3)	0.448
III	4 (9)	20 (13.6)	0.402
**Tumour characteristics**			
More than three lesions	3 (7)	18 (12.2)	0.297
Tumour size ≥ 5 cm	12 (27)	28 (19.0)	0.273
Bi-lobar disease	13 (29)	54 (36.7)	0.315
Synchronous disease	22 (49)	89 (60.5)	0.157
Lymph node-positive	27 (60)	113 (76.8)	0.035
Extrahepatic disease	8 (18)	36 (24.5)	0.310
**Perioperative outcomes**			
Major resection	14 (31)	54 (36.7)	0.490
Median (range) duration of surgery (min)	292	150	0.002
Median (range) intraoperative blood loss (ml)	200	100	0.002
Pringle’s manoeuvre	0	28 (19.0)	0.002
Conversion	8 (18)	9 (6.1)	0.016
Postoperative complications	11 (24)	24 (16.3)	0.219
Clavien-Dindo above III	7 (16)	12 (8.1)	0.146
Bleeding	9 (20)	9 (6.1)	0.005
Reoperation	3 (7)	1 (0.7)	0.015
Blood transfusion	12 (27)	18 (12.2)	0.020
Bile leak	1 (2)	5 (3.4)	0.016
Median (range) duration of hospital stay (day)	7 (2–147)	5 (1–112)	0.002
**Oncological outcomes**			
R1	0 (0)	15 (10.2)	0.026
Median length of follow-up (months)	44	34	0.061
**Median OS (months)**	45	60	0.286
1 year	91%	96%	
5 years	47%	52%	
10 years	23%	36%	
**Median RFS (months)**	28	15	0.760
1 year	75%	58%	
5 years	33%	33%	

Data are *n* (%) unless otherwise stated. EPc, early-phase cohort; LPc, late-phase cohort; OS, overall survival; RFS, recurrence-free survival.

Overall, an R0 resection was achieved in 93 per cent (100 per cent in the EPc and 90 per cent in the LPc; *P* = 0.026). Survival analysis of the whole CRLM cohort with a median follow-up of 37 months revealed a median OS of 60 months; the 1-, 3-, 5-, and 10-year survival rates were 95 per cent, 66 per cent, 51 per cent, and 29 per cent, respectively. The median RFS was 16 months; the 1-, 3-, and 5-year RFS rates were 63 per cent, 38 per cent, and 33 per cent, respectively.

Survival analysis comparison between the EPc and LPc groups showed no impact of the LC on OS (hazard ratio (h.r.) 0.78, 95 per cent confidence interval (c.i.) 0.51 to 1.2; *P* = 0.286). The 5-year OS rate was 47 per cent for the EPc group *versus* 52 per cent for the LPc group. Similarly, the LC did not affect RFS (h.r. 0.94, 95 per cent c.i. 0.64 to 1.37; *P* = 0.760). The 3- and 5-year RFS rates were, respectively, 42 per cent and 33 per cent for the EPc group *versus* 37 per cent and 33 per cent, respectively, for the LPc group.

After applying propensity scores to match patients in the EPc group at a ratio of 1:1 with those in the LPc group, (EPcm and LPcm) the R1 resection, median OS, and RFS were similar (*Table 3*).

**Table 3:** Colorectal liver metastasis propensity score-matched groups: patient characteristics and oncological data

	EPcm (*n* = 45)	LPcm (*n* = 45)	*P*	SMD
**Patient characteristics**				
** **Median (range) age (years)	65 (27–77)	66 (23–84)	0.670	
Age > 70 years	11 (24)	11 (24)	1	0
Male sex	26 (58)	23 (51)	0.531	0.067
ASA				
I	8 (18)	9 (20)	0.787	0.022
II	33 (73)	35 (78)	0.623	0.045
III	4 (9)	1 (2)	0.167	0.067
**Tumour characteristics**				
More than three lesions	3 (7)	3 (7)	1	0
Tumour size ≥ 5 cm	12 (27)	9 (20)	0.460	0.067
Bi-lobar disease	13 (30)	14 (31)	0.821	0.02
Synchronous disease	22 (49)	28 (62)	0.207	0.13
Lymph node-positive	27 (60)	31 (69)	0.378	0.09
Extrahepatic disease	8 (18)	9 (20)	0.712	0.02
**Perioperative outcomes**				
Major resection	14 (31)	13 (29)	0.818	0.022
Postoperative complications	11 (24)	9 (20)	0.617	0.044
Clavien-Dindo > III	7 (16)	4 (9)	0.330	0.67
Bleeding	9 (20)	5 (11)	0.240	0.089
Reoperation	3 (7)	0 (0)	0.085	0.067
Blood Transfusion	12 (27)	7 (16)	0.196	0.112
Bile leak	1 (2)	1 (2)	1	0
Median duration of hospital stay (days)	7 (2–147)	5 (1–112)	0.031	
**Oncological outcomes**				
R1	0 (0)	1 (2)	0.320	0.022
Median follow-up (months)	44	44	1	
**Median OS (months)**	45	49	0.580	
1 year	91.1%	91.1%		
5 years	44.8%	42.8%		
10 years	23.2%	34.8%		
**Median RFS (months)**	23	16	0.433	
1 year	71.1%	53.3%		
5 years	27.0%	34.3%		

Data are *n* (%) unless otherwise stated. EPcm, early-phase matched group; LPcm, late-phase matched group; OS, overall survival; RFS, recurrence-free survival.

## Discussion

This paper reports the experience of a pioneer surgeon in minimally invasive surgery who has performed many complex laparoscopic gastrointestinal procedures since the 1990s and has experience in open liver resection before progressing to minimally invasive counterparts. In 2002, the first laparoscopic right hepatectomy was performed. This was done on the fourth patient of the cohort, and the surgery was converted to open due to bleeding. The first successful laparoscopic right hepatectomy was done in 2003. As both major and minor liver resections were started simultaneously, it was elected to establish one LC based on estimated blood loss and conversion rate for both. The data showed that 68 cases were required before competence was attained. As expected, conversion for bleeding control declined with increasing experience and confidence in laparoscopic haemostasis. The use of the Pringle manoeuvre with CUSA (Cavitron Ultrasonic Surgical Aspirator; Valleylab, Boulder, CO, USA) allowed for meticulous dissection in an almost bloodless field. In addition, port positions were refined to foster ergonomics for parenchymal transection and intracorporeal suturing. The establishment of a consistent theatre team trained in both open liver surgery and laparoscopic surgery was also a feature of this series. The overall conversion rate was 9 per cent. This was 19 per cent in the EP and decreased to 6 per cent in the LP, consistent with the literature, which reported an overall conversion rate between 4 and 17 per cent; this varies according to the complexity of LLR^8,9,11,13,14^.

Previously published studies suggest that laparoscopic resection for CRLM is oncologically equivalent to open surgery and does not compromise oncological long-term outcomes^2,3,15–25^. The recently published OSLO-COMET trial, which compared OLR and LLR for parenchyma-sparing liver resection, and the LapOpHuva trial, which included major resections, showed that LLR did not impact on survival for CRLM^26,27^. It is worth noting that the OSLO-COMET trial was started after at least 400 LLRs were performed in the centre. Similarly, the LapOpHuva trial was conducted after more than 50 cases had been performed. This leaves the question of whether the LC influences patient survival unanswered.

Kim *et al*. studied the impact of LC on the survival of gastric cancer, finding improved outcomes with increased surgeon experience^28^. This was attributed to a higher number of lymph nodes harvested by experienced surgeons. In another study by Park *et al*. on the effect of the LC on survival after colonic surgery, the LC impacted on survival only in stage III disease, where nodal metastasis and hence lymphadenectomy would affect survival^29^. In liver resections, a positive margin is the main surgical factor associated with poor survival outcomes. There were no positive resection margins in the EPc group. This could have contributed to the lack of effect of the LC on survival outcomes. The lack of R1 reflects the careful patient selection and approach taken to delineate intrahepatic tumour anatomy with the aid of intraoperative ultrasound to overcome the limitation created by the loss of tactile feedback. In LPc, the indications were extended, and a higher proportion of patients underwent resection for tumours with proximity to major blood vessels and the resection plane. This resulted in a higher R1 resection rate of 10 per cent. The overall R1 (7 per cent) rate in the present series compares favourably with the literature, which reports an incidence rate of between 5 and 24 per cent for OLR and between 1 and 12 per cent for LLR^15,18,20,30–33^.

Analysis of the CRLM cohort did not show a significant difference in OS or RFS between the two phases of the LC. Variability in the selection criteria for resection over time was anticipated, and this was revealed by a higher proportion of lymph node-positive primary tumour in the LPc group. It is well known that factors including tumour dimension larger than 5 cm, multiple liver lesions, bi-lobar liver disease, synchronous liver metastasis, lymph node-positive primary tumour, and the presence of extrahepatic disease poorly affect survival for CRLM^24,34^. Therefore, a 1:1 propensity score matching was applied to create two comparable groups. Survival analysis of the balanced cohort again showed no effect of the LC on OS or RFS.

Certain limitations of this study should be acknowledged. Firstly, it was a retrospective study from a prospective database. Secondly, this LC would be different for other surgeons who develop their laparoscopic skills by doing LLRs. Thirdly, we did not study the effect of chemotherapy, which may have resulted in improved survival outcomes over the last decade^15,17,35,36^.

*Disclosure*. The authors declare no conflict of interest.

## Data Availability

Participants of this study did not agree for their data to be shared publicly, so supporting data are not available.
